# Experimental Warming Increased Cooked Rice Stickiness and Rice Thermal Stability in Three Major Chinese Rice Cropping Systems

**DOI:** 10.3390/foods13111605

**Published:** 2024-05-22

**Authors:** Huifang Yang, Liming Chen, Ruoyu Xiong, Yanhua Zeng, Yu Jiang, Jun Zhang, Bin Zhang, Taotao Yang

**Affiliations:** 1 Rice Research Institute, Guangdong Academy of Agricultural Sciences, Guangzhou 510640, China; 2 Key Laboratory of Plant Molecular Physiology, Institute of Botany, The China Academy of Science, Beijing 100093, China; 3 Jiangxi Key Laboratory of Plant Resources and Biodiversity, Jingdezhen University, Jingdezhen 333400, China; 4 Ministry of Education and Jiangxi Key Laboratory of Crop Physiology, Ecology and Genetic Breeding, Jiangxi Agricultural University, Nanchang 330045, China; 5 Jiangsu Collaborative Innovation Center for Modern Crop Production, Nanjing Agricultural University, Nanjing 210095, China; 6 Institute of Crop Sciences, Chinese Academy of Agricultural Sciences, Beijing 100081, China

**Keywords:** climate warming, cropping systems, rice quality, starch structure

## Abstract

Climate warming is a critical environmental issue affecting rice production. However, its effects on cooked rice texture and rice thermal properties remain unstudied in China. To address this gap, we conducted a two-year multi-site field warming experiment using free-air temperature increase facilities across three major Chinese rice cropping systems. Interestingly, warming had a minimal impact on the hardness of cooked rice, while it significantly increased stickiness by an average of 16.3% under warming conditions. Moreover, compared to control treatments, rice flour exhibited a significant increase in gelatinization enthalpy, onset, peak, and conclusion temperatures under warming conditions, with average increments of 8.7%, 1.00 °C, 1.05 °C, and 1.17 °C, respectively. In addition, warming significantly declined the amylose content, remarkedly elevated the protein content and relative crystallinity, and altered the weight distribution of the debranched starch. Correlation analysis revealed significant relationships between cooked rice stickiness, rice flour thermal properties, amylose content, protein content, and partial starch structures. Therefore, warming-induced alterations in rice composition and starch structure collectively enhanced cooked rice stickiness and rice thermal stability.

## 1. Introduction

Rice is one of the most crucial cereal grains around the globe, serving as a principal source of energy and nutrients for humans. China, which is the largest consumer and producer of rice worldwide, reported a rice planting area covering 30.0 million square kilometers in 2021, yielding a total grain output of approximately 211.9 million tons [1]. With predictions suggesting a 1.6 °C to 2.5 °C rise in the global surface temperature between 2041 and 2060 compared to that between 1850 and 1900 [2], climate warming is inevitable, posing numerous challenges to rice production in China [3].

As living standards improve, there is an increasing demand for high-quality rice, particularly with regard to cooking and eating attributes. Temperature has emerged as a crucial abiotic factor influencing rice quality. To examine the effect of climate warming on rice quality, a series of temperature control experiments have been carried out [4,5]. Free-air temperature increase (FATI) facilities, which emit infrared radiation directly onto rice plants, offer a controlled warming environment under natural wind and light conditions [6]. FATI facilities have been proven to be instrumental in simulating future global warming scenarios and are extensively utilized in field warming experiments [7,8,9]. Over the past few years, numerous studies employing FATI facilities have explored the influence of climate warming on rice milling, appearance, cooking, and eating qualities. For instance, prior research demonstrated that warming detrimentally affects the milling and appearance qualities of rice while also altering its pasting properties in middle rice cropping systems (CS_S_) [7,8,10]. In double rice CS_S_, our prior studies revealed that warming alters rice milling quality, appearance quality, and pasting properties, with variations observed between early and late seasons and among varieties [9,11,12].

The gelatinization of rice flour is a crucial physicochemical characteristic for evaluating rice eating and cooking qualities. Researchers utilize differential scanning calorimetry (DSC) to examine the thermal properties of rice, including the gelatinization enthalpy and gelatinization temperatures. Rice starch with lower gelatinization temperatures and a lower gelatinization enthalpy typically requires less time and heat for cooking [13]. In addition, thermal parameters significantly influence cooked rice texture and sensory perception [14,15]. Since rice is predominantly consumed in its cooked form, textural attributes such as stickiness and hardness directly impact its eating quality and consumer acceptance [16]. To our knowledge, research on the impact of warming on rice cooking and eating qualities, particularly regarding cooked rice texture and thermal properties, using FATI facilities is limited [17]. Therefore, it is imperative to clarify the impact of warming on cooked rice texture and rice thermal properties in China.

Rice starch comprises two major biomacromolecules, amylose and amylopectin, constituting roughly 90% of the grain weight. These components are pivotal factors contributing to rice quality [18]. In addition to rice components, multi-level starch structures, including starch molecular structures, crystalline structures, and starch–protein complexes, collectively regulate rice quality [19]. Although numerous studies have investigated the relationships between rice appearance quality, pasting properties, rice components, starch crystalline structure, and amylopectin molecular structure under warming conditions [20,21,22], the impact of rice components and multi-level starch structures on the textural and thermal properties of Chinese rice under field warming conditions remains uncertain.

The cultivation of rice in China primarily occurs across three main cropping regions: double rice (early rice and late rice) in the South, middle rice in East and Central China, and single rice in the Northeast, collectively accounting for 96% of the country’s rice cultivation [23]. Previous research has demonstrated that climate warming impacts rice growth and grain yield in the three major rice production systems, with significant variations observed among the three regions [3,23]. Therefore, we hypothesized that warming would alter the textural and thermal properties of rice in three major Chinese rice CS_S_. Our primary objective was to evaluate the impacts of warming on cooked rice texture and rice thermal properties, as well as their differences among the three major Chinese rice CS_S_, and to preliminarily explore the mechanism of quality change from the aspects of rice components and multi-level starch structures. To achieve this goal, two-year field warming experiments were carried out using FATI facilities in single rice CS_S_, middle rice CS_S_, and double rice CS_S_.

## 2. Materials and Methods

### 2.1. Site Description

In 2021 and 2022, we carried out field warming experiments in single rice CS_S_ in Harbin (126°48′ E, 45°49′ N), middle rice CS_S_ in Zhenjiang (119°28′ E, 31°54′ N), and double rice CS_S_ in Guangzhou (113°32′ E, 23°09′ N) (Figure S1). Each experimental site has its own unique climate conditions. Harbin has a temperate continental monsoon climate, Zhenjiang has a subtropical monsoon climate, and Guangzhou has a tropical monsoon climate. Before commencing the experiments, the topsoil properties (0–15 cm) were as follows: pH 7.8, 1.1 g kg^−1^ total nitrogen, and 16.7 g kg^−1^ soil organic carbon in Harbin; pH 6.3, 1.1 g kg^−1^ total nitrogen, and 21.1 g kg^−1^ soil organic carbon in Zhenjiang; and pH 5.9, 1.1 g kg^−1^ total nitrogen, and 25.3 g kg^−1^ soil organic carbon in Guangzhou.

### 2.2. Crop Management

In this study, the rice cultivars used were Longdao18 (inbred *japonica* rice) for single rice CS_S_, Wuyunjing23 (inbred *japonica* rice) for middle rice CS_S_, and Yuehesimiao (inbred *indica* rice) for double rice CS_S_. The rice seedlings were transplanted manually. In single rice CS_S_, the spacing between hills was 25.0 cm × 10.0 cm, with 5 seedlings in each hill. In middle rice CS_S_, the spacing between hills was 25.0 cm × 15.0 cm, with 3 seedlings in each hill. Finally, in double rice CS_S_, the spacing between hills was 19.8 cm × 16.5 cm, with 2 or 3 seedlings in each hill. Table S1 provides an overview of the rice phenology observed in 2021 and 2022. Urea (with 46% nitrogen content), calcium magnesium phosphate (containing 12.0% P_2_O_5_), and potassium chloride (with 60% K_2_O) were utilized as nitrogen, phosphorus, and potassium fertilizers, respectively. For the three experimental sites, the application rates and management practices of fertilizers are shown in Table S2. Additionally, we adhered to local high-yield cultivation practices for other cultivation methods and field management.

### 2.3. Experimental Design and Field Warming System

The field experiments at the three experimental sites involved a randomized complete block design with 3 replicates, comprising both a whole-growth-period canopy warming treatment (Warming) and an ambient temperature treatment (Control). For the warming treatments, we utilized FATI facilities to elevate the canopy temperature of rice plants from transplanting to maturity. Specifically, we suspended an infrared heater (20 cm width, 180 cm length, and 1500 W) at a distance of 0.75 m above the midpoint of the last leaf in each warming treatment. The heater’s height was adjusted as the growth period advanced. To mitigate the shading impact of the heater, we also suspended a ‘dummy’ heater at the same distance in each control treatment. In addition, a temperature sensor was suspended in the midpoint of the last leaf in each treatment. Continuous monitoring of the rice canopy temperatures was conducted at hourly intervals. In general, the temperature trend of the rice canopy was not altered by the FATI facilities (Figure S2). Warming treatments exhibited an increase in canopy temperature of 1.7–1.8 °C for single rice, 2.0–2.5 °C for middle rice, 1.8–2.0 °C for early rice, and 2.0 °C for late rice, compared to control treatments (Table S3). The FATI facilities successfully induced the expected temperature rise, aligning with our research objectives.

### 2.4. Measurements

#### 2.4.1. Sample Preparation

After reaching maturity, the rice grains were collected using a portable thresher. Subsequently, the rice grains were air-dried and kept at room temperature for 3 months. Following the storage period, the rice grains were milled using a rice polishing machine (LTJM-5588, Taizhou Grain Instrument Factory, Taizhou, China), and then the broken rice was separated from the head rice. To obtain a sample of rice flour, approximately 20 g of head rice was ground into flour, followed by passing it through a 0.25 mm sieve.

For the preparation of rice starch samples, a modified version of our previous method was employed [24]. First, 10 g of head rice was soaked in an 80 mL solution containing 10 mg g^−1^ alkaline protease and 0.45% sodium metabisulfite, which was maintained at 37 °C overnight. After soaking, the rice was ground into a slurry and sieved through a 200 mesh. The obtained filtrate underwent centrifugation (3000× *g*, 10 min) after being stored at 37 °C overnight, then the supernatant was discarded. This steeping and centrifugation process was repeated 5 times. Lastly, the starch was air-dried at 40 °C, ground, and sieved through a 200 mesh.

#### 2.4.2. Assessment of Thermal Properties

Differential scanning calorimetry (DSC Q2000, TA Instruments, New Castle, DE, USA) was utilized to examine the thermal properties of the rice flour following the methods described in our previous study [24]. Specifically, 5 mg of head rice flour was added into 10 mL ultrapure water and sealed in an aluminum pan at 4 °C overnight. Subsequently, the sealed sample pans, along with an empty sealed reference pan, were heated from 20 °C to 100 °C at 10 °C min^−1^ within the DSC chamber. Each sample was tested twice to ensure accuracy.

#### 2.4.3. Assessment of Cooked Rice Texture

A 20 g sample of head rice underwent washing 3 times in an aluminum can. Following this, the rice-to-water weight ratio was adjusted to 1:1.3 by adding ultrapure water. Next, the sealed aluminum can with filter paper was positioned on a steaming tray and cooked for 30 min. After cooking, the rice sample was allowed to cool for another 30 min. Three complete grains were selected from the cooked rice and then placed on the testing platform in a triangular shape. Texture analysis was carried out using a texture analyzer equipped with an aluminum alloy cylindrical probe (TVT 6700, Perten, Hagersten, Sweden), executing a compressed twice program. The parameters for the texture analyzer were as follows: pre-test, test, and post-test speed of 1.0 mm s^−1^, and compression strain of 60%. From the obtained texture profile, the stickiness and hardness of the cooked rice were assessed. Each treatment involved the cooking of two rice samples, with each cooked rice sample undergoing six measurements to ensure reproducibility.

#### 2.4.4. Analysis of Total Starch, Amylose, Amylopectin, and Protein Contents

The total starch, amylose, and amylopectin contents of head rice flour were assessed using a total starch, amylose, and amylopectin assay kit following the manufacturer’s instructions (Suzhou Komin Biotechnology Co., Ltd., Suzhou, China). Meanwhile, the protein content of head rice flour was determined using a Kjeldahl nitrogen analyzer (Kjeltec 8400, FOSS, Hilleroed, Denmark) to measure the nitrogen content, which was then multiplied by a conversion factor of 5.95 [9].

#### 2.4.5. Analysis of X-ray Diffraction

X-ray diffractograms of head rice flour were obtained using an X-ray powder diffractometer (X’Pert Pro, PANalytical, Almelo, The Netherlands) following the methodology established in our previous study [24]. The diffractometry analysis was conducted at 40 kV and 200 mA, with a diffraction angle (2θ) of 3° to 40°, a speed of 0.02°, and an interval of 0.6 s. Each sample underwent duplicate assays to ensure accuracy. Subsequently, MDI-Jade-6 software was employed to compute the relative crystallinity percentage according to the acquired results.

#### 2.4.6. Analysis of the Weight Distribution of Debranched Starch

The isolated starch (5 mg) was dissolved in 0.9 mL water in a boiling water bath for 15 min. Following dissolution, the starch dispersion underwent a 3 h incubation at 37 °C in a water bath. To the dispersion, isoamylase (1400 U, 10 μL), acetate buffer (0.1 M, 0.1 mL, pH 3.5), and sodium azide solution (40 mg mL^−1^, 5 mL) were added before the incubation. The resulting mixture was then precipitated using 5 mL of absolute ethanol, followed by centrifugation at 4000× *g* for 10 min. The debranched starch was subsequently redissolved in 1 mL DMSO/LiBr solution for 2 h at 80 °C.

To assess the molecular weight distribution of debranched starch, a gel permeation chromatography system (AcQuity UPLC, Waters, Milford, MA, USA) equipped with a differential refractive index detector and two tandem columns was utilized. Data acquisition and processing were performed using ASTRA 6.1, with technical support from Sanshu Biotech. Co., Ltd. (Nanjing, China, http://www.sanshubio.com/starch-molecular-weight-distribution, accessed on 25 December 2022).

### 2.5. Statistical Analysis

All the data underwent analysis with SPSS v25.0 software (SPSS, Inc., Chicago, IL, USA). For each CS, a general linear model analysis of variance was conducted to ascertain the potential significance and interactions between “year” and “treatment”. The means of different treatments within each CS were compared using Student’s *t*-test at the 0.01 and 0.05 significance levels. Pearson correlation analysis was employed to explore correlations among rice components, starch structures, cooked rice texture, and thermal properties at the 0.01 and 0.05 significance levels.

## 3. Results and Discussion

### 3.1. Impact of Experimental Warming on Rice Components, Weight Distribution of Debranched Starch, and Relative Crystallinity

**Figure 1:**
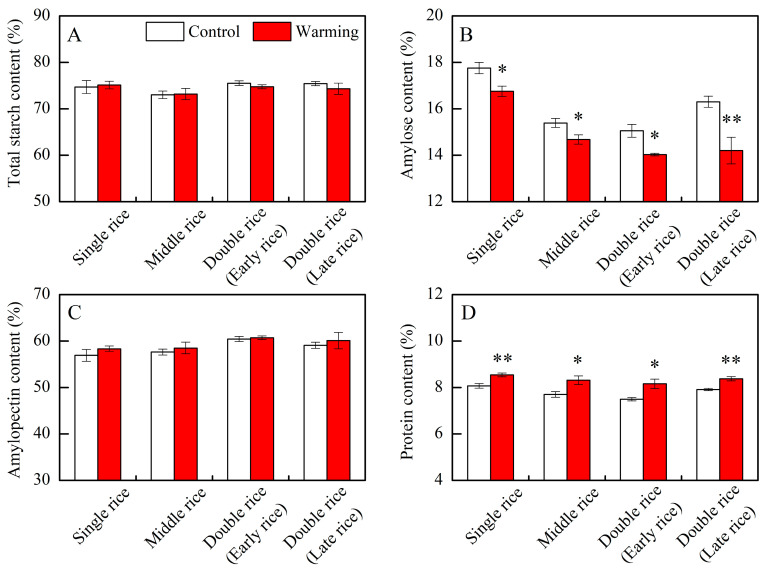
Effects of experimental warming on rice components in three major Chinese rice cropping systems. Control, ambient temperature treatment; Warming, whole-growth-period warming treatment. (**A**) total starch content; (**B**) amylose content; (**C**) amylopectin content; (**D**) protein content. Values were averaged over two years. Error bars represent the standard deviation of the two-year mean. * and ** indicate significant differences between treatments over two years at the *p* < 0.05 and *p* < 0.01 levels, respectively.

In this research, warming showed a notable impact on the amylose and protein levels of head rice across three major Chinese rice CS_S_, while it did not significantly affect the total starch and amylopectin contents (Table 1). Compared to control treatments, the amylose contents decreased by an average of 1.02, 0.71, 1.00, and 2.10 percentage points in single, middle, early, and late rice, respectively, under warming conditions (Figure 1B). Conversely, protein contents increased by an average of 0.47, 0.61, 0.67, and 0.46 percentage points in the respective CS_S_ (Figure 1D). Our findings align closely with prior studies, indicating a consistent trend of decreased amylose content and increased protein content in rice under warming conditions in middle rice CS_S_ [8,21,25]. Previous research suggests that the decline in amylose content may be related to reduced granule-bound starch synthase activity [26], while the rise in protein content may be associated with increased activities of glutamine synthetase and glutamate synthase [10,27]. Amylose and protein are important components of rice, which directly affect its cooking and eating qualities. Within a certain range, amylose and protein contents are significantly negatively correlated with the taste value of *indica* rice [28]. In this study, the decreased amylose content and increased protein content in rice under warming conditions might have led to complex changes in the taste value.

**Figure 2:**
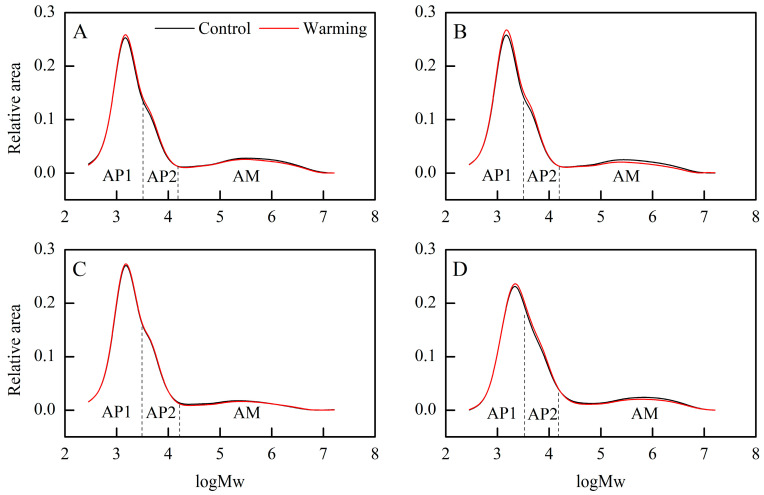
Effects of experimental warming on the weight distribution of debranched starch in three major Chinese rice cropping systems (2022). Control, ambient temperature treatment; Warming, whole-growth-period warming treatment. (**A**) Single rice; (**B**) middle rice; (**C**) double rice (early rice); (**D**) double rice (late rice). AP1, amylopectin short branch chains; AP2, amylopectin long branch chains; AM, amylose chains.

Furthermore, we examined the weight distribution of debranched starch and identified three categories: amylopectin short branch chains (AP1), amylopectin long branch chains (AP2), and amylose chains (AM) [29]. Warming induced changes in the weight distribution of debranched starch (Figure 2), with increases in the relative areas of AP1 and AP2, particularly notable in single, middle, and late rice (Table 2). Conversely, the relative AM area decreased significantly across all CS_S_ under warming conditions, with an average decrease of 2.22 percentage points (Table 2). In contrast, previous research found that high temperatures might decrease AP1 while increasing AP2 [20,30]. These changes in the weight distribution of debranched starch under warming conditions indicate alterations in starch synthesis pathways [31]. The synthesis of starch in the rice endosperm involves various key enzymes, such as granule-bound starch synthase, adenosine diphosphate glucose pyrophosphorylase, and soluble starch synthase [32]. The complex and coordinated influence of temperature on these enzymes necessitates further investigation to elucidate the underlying mechanisms [32].

**Figure 3:**
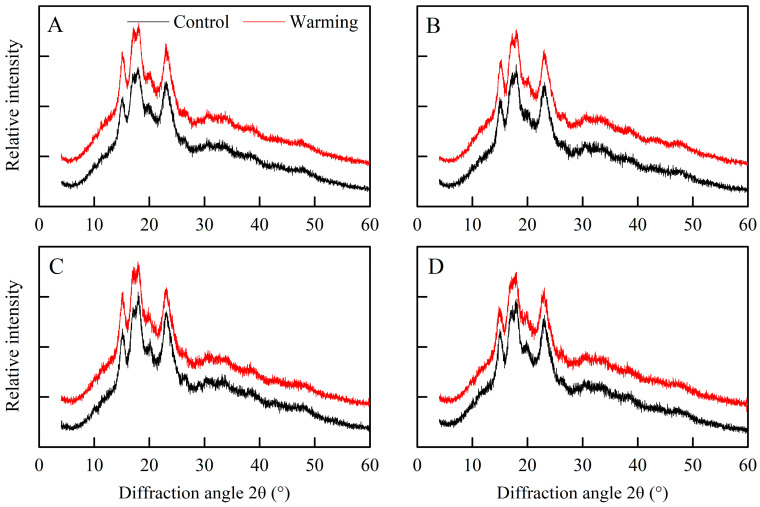
Effects of experimental warming on the X-ray diffractograms of rice flour in three major Chinese rice cropping systems (2022). Control, ambient temperature treatment; Warming, whole-growth-period warming treatment. (**A**) Single rice; (**B**) middle rice; (**C**) double rice (early rice); (**D**) double rice (late rice).

Based on the findings shown in Figure 3, all the rice flour samples displayed a distinctive A-type diffraction pattern, with five prominent peaks at 15°, 17°, 18°, 20°, and 23° under both warming and control conditions [33]. However, there were notable differences in the relative crystallinity of rice flour between the warming and control treatments (Table 2). Specifically, the relative crystallinity of single, early, and late rice was markedly elevated under warming conditions compared to the control treatments, with average increments of 1.10, 1.19, and 1.15 percentage points, respectively. Similarly, a previous study demonstrated that warming through FATI facilities raised the relative crystallinity of rice flour in middle rice CS_S_ [21].

The crystallinity of rice starch relies on the amylopectin skeleton, with the short branch chains of amylopectin contributing to crystalline formation [13]. Moreover, amylose molecules predominantly exist as amorphous matter within starch granules and are believed to introduce defects in amylopectin crystals [29]. Hence, the higher relative crystallinity of rice flour under warming conditions was due to the reduced amylose content and the increased relative area of AP1. Taken together, warming induced changes in the rice components, crystalline structure, and molecular structure of rice starch in three major Chinese rice CS_S_. In the following section, we explore how these alterations impact cooked rice texture and rice thermal properties.

### 3.2. Effect of Experimental Warming on Cooked Rice Texture

**Figure 4:**
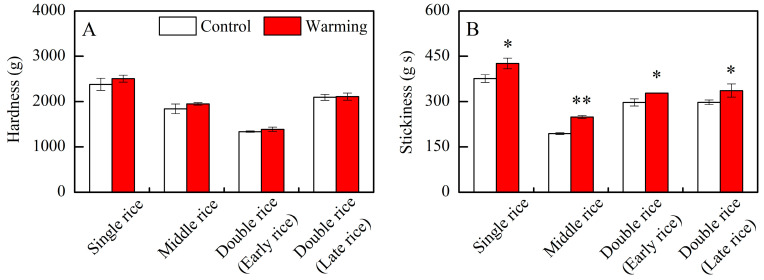
Effects of experimental warming on cooked rice texture in three major Chinese rice cropping systems. Control, ambient temperature treatment; Warming, whole-growth-period warming treatment. (**A**) Hardness; (**B**) stickiness. Values were averaged over two years. Error bars represent the standard deviation of the two-year mean. * and ** indicate significant differences between treatments over two years at the *p* < 0.05 and *p* < 0.01 levels, respectively.

In the three major Chinese rice CS_S_, warming did not have an obvious impact on the hardness of cooked rice; however, there were notable differences in the stickiness of cooked rice between warming and control treatments (Table 1). Warming led to obvious increases in the stickiness of single, middle, early, and late rice by an average of 13.3%, 28.1%, 10.6%, and 13.3%, respectively, compared to the control treatments (Figure 4B). Generally, Chinese consumers prefer cooked rice with lower firmness and stickier texture within a certain range [34,35]. In this study, warming elevated the stickiness of cooked rice across three major Chinese rice CS_S_, potentially indicating improved palatability under future climate warming conditions. Similarly, Jing et al. [22,36] reported that warming, achieved by emitting heat radiation from running water in tubes, increased the stickiness and taste value of cooked rice under field conditions in a middle rice CS_S_. However, Chun et al. [20] reported that the taste value and palatability of rice grown at high temperatures in greenhouses decreased without affecting the hardness and stickiness of cooked rice. Therefore, the effects of warming or high temperature on cooked rice texture vary greatly among studies and might be related to changes in rice components and starch structures.

**Figure 5:**
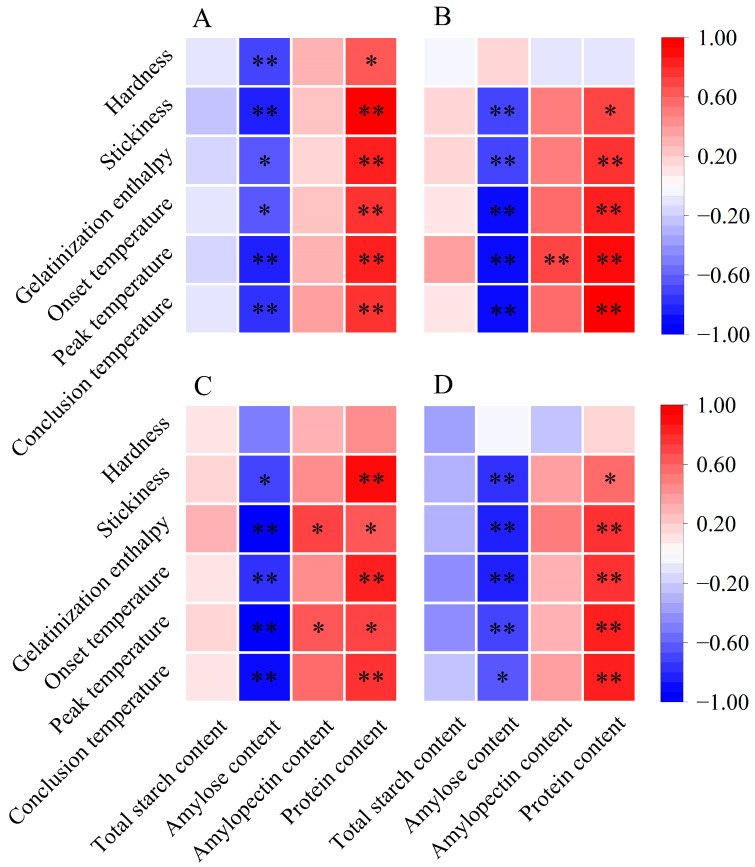
Pearson correlation analysis between rice components, cooked rice texture, and thermal properties in three major Chinese rice cropping systems (*n* = 12). * and ** indicate significant correlations at the *p* < 0.05 and *p* < 0.01 levels, respectively. (**A**) Single rice; (**B**) middle rice; (**C**) double rice (early rice); (**D**) double rice (late rice).

**Figure 6:**
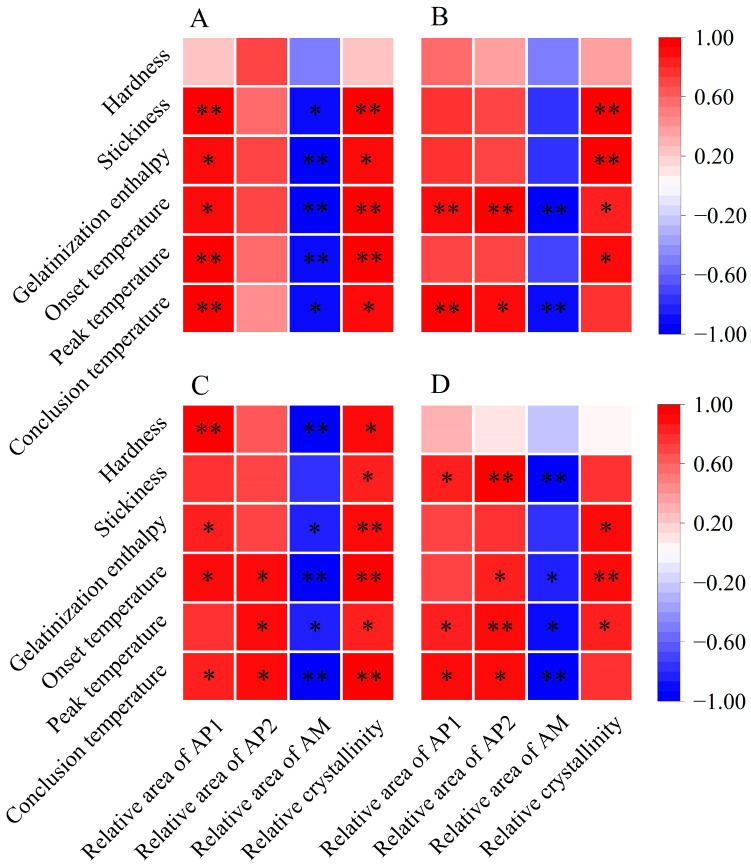
Pearson correlation analysis between starch structures, cooked rice texture, and thermal properties in three major Chinese rice cropping systems (*n* = 6). * and ** indicate significant correlations at the *p* < 0.05 and *p* < 0.01 levels, respectively. (**A**) Single rice; (**B**) middle rice; (**C**) double rice (early rice); (**D**) double rice (late rice). AP1, amylopectin short branch chains; AP2, amylopectin long branch chains; AM, amylose chains.

Previous research has shown that the stickiness of cooked rice is inversely related to the amylose content and is positively associated with the amylopectin content [16,37]. Additionally, the molecular structure of amylopectin influences the stickiness of cooked rice. According to Li and Gilbert [16], the increased proportion of amylopectin short branch chains fosters bonding and molecular interactions, necessitating more force to separate grains and resulting in increased stickiness. Herein, we found a significant negative correlation between the stickiness of cooked rice and its amylose content (Figure 5). Furthermore, the relative area of AP1 was positively associated with stickiness, especially for single rice and late rice, demonstrating significant positive correlations with stickiness (Figure 6). Therefore, the elevated stickiness of cooked rice in three major Chinese CS_S_ under warming conditions might be due to a reduction in amylose content and an increase in the relative area of AP1.

Apart from the rice components and starch structure, the chemical and physical characteristics of leached materials that adhere to the surface of cooked rice grains also influence their stickiness [38]. The stickiness of cooked rice is positively associated with the quantity of leached amylopectin and the proportion of amylopectin short branch chains in these materials [16]. Increased leached amylopectin and a higher proportion of amylopectin short branch chains promote better bonding conditions and stronger intermolecular interactions, thereby enhancing stickiness [14,16]. However, we found that warming had no effect on the total amount or components of leachate during the cooking process (Table S4). Thus, the impacts of warming on the molecular structure of leached starch and its influence on the stickiness of cooked rice need to be further clarified.

Generally, the hardness of cooked rice is thought to be inversely proportional to its stickiness [16]. However, this study diverges from this notion, revealing that the hardness of cooked rice in three major Chinese CS_S_ remained unchanged under warming conditions, despite a significant increase in the stickiness of cooked rice (Figure 4). The hardness of cooked rice is typically associated with the amylose content [16,37], as amylose molecules can impede starch granule swelling during cooking [35,37]. In addition to rice components, amylopectin long branch chains also affect the hardness of cooked rice by interacting with other components in rice grains, such as lipids and proteins, restricting starch swelling and resulting in a firmer texture [34,37]. Herein, we observed a significant decrease in amylose content, a significant increase in protein content, and an increasing trend in the relative area of AP2 under warming conditions (Figure 1B,D; Table 2). Therefore, the impact of warming on rice components and starch molecular structure appears intricate, possibly counterbalancing the effects of warming on the hardness of cooked rice.

### 3.3. Effects of Experimental Warming on Rice Thermal Properties

**Figure 7:**
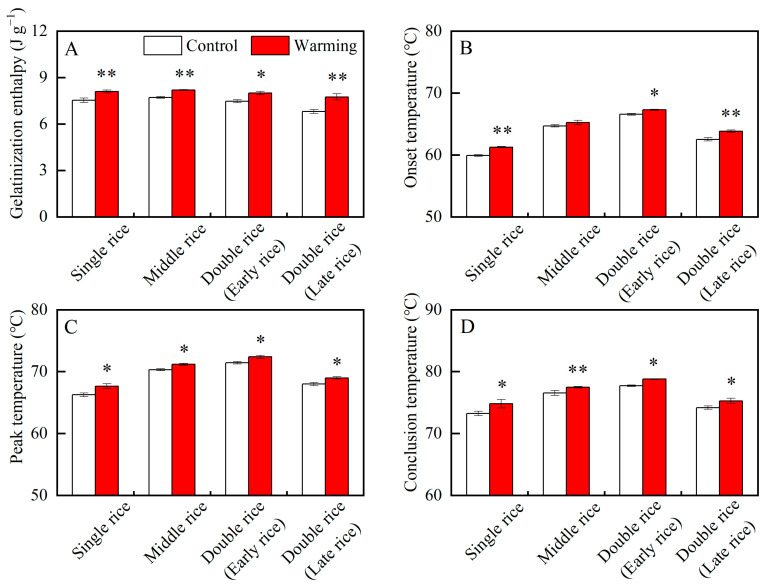
Effects of experimental warming on the thermal properties of rice flour in three major Chinese rice cropping systems. Control, ambient temperature treatment; Warming, whole-growth-period warming treatment. (**A**) Gelatinization enthalpy; (**B**) onset temperature; (**C**) peak temperature; (**D**) conclusion temperature. Values were averaged over two years. Error bars represent the standard deviation of the two-year mean. * and ** indicate significant differences between treatments over two years at the *p* < 0.05 and *p* < 0.01 levels, respectively.

The thermal properties of rice are closely related to its cooking and eating qualities [30]. As shown in Table 1, except for the onset temperature of middle rice, the gelatinization enthalpy and gelatinization temperatures significantly varied between the warming and control treatments in three major Chinese rice CS_S_. On average, across years, warming led to obvious increases in the gelatinization enthalpy of single, middle, early, and late rice by 7.57%, 6.16%, 7.19%, and 13.86%; the onset temperature of single, early, and late rice by 1.37 °C, 0.73 °C, and 1.33 °C; the peak temperature of single, middle, early, and late rice by 1.38 °C, 0.88 °C, 0.95 °C, and 1.00 °C; and the conclusion temperature of single, middle, early, and late rice by 1.58 °C, 0.93 °C, 1.07 °C, and 1.08 °C, respectively (Figure 7). Similar results were found in a previous study, in which elevated temperature increased the gelatinization enthalpy and temperatures of middle rice flour [22]. Higher gelatinization enthalpies and temperatures reflect the higher cooking temperature and longer cooking time required for head rice [30]. Herein, the head rice in three major Chinese rice CS_S_ required more energy to cook as it matured under warming conditions. These findings pose challenges for sustainable rice production and environmental enhancement. In addition, previous studies have shown that high temperature leads to higher gelatinization temperatures and gelatinization enthalpy of starch and simultaneously reduces the taste value and palatability of cooked rice [20,30]. Therefore, from the perspective of gelatinization properties, warming may lead to poorer eating quality of rice in three major Chinese rice CS_S_.

The variations in thermal parameters reflect a complex interplay of factors, such as amylose content, amylopectin content, starch molecules, and crystalline structures [13,19]. A higher gelatinization enthalpy indicates a greater number of ordered crystalline structures in rice starch [13,39]. As previously discussed, amylose is negatively correlated with relative crystallinity, while amylopectin plays an essential role in determining the relative crystallinity of starch [13,29]. Notably, an abundance of amylopectin short branch chains can contribute to the formation of an ordered crystalline structure [34,39]. In our study, we observed a negative correlation between amylose content and gelatinization enthalpy across three major Chinese CS_S_ (Figure 5). Conversely, gelatinization enthalpy showed a significant positive correlation with relative crystallinity (Figure 6). Additionally, the relative area of AP1 demonstrated a positive correlation with gelatinization enthalpy, particularly notable for single and early rice (Figure 6). Therefore, all rice in the three major rice CS_S_ exhibited increased gelatinization enthalpy under warming conditions, likely due to their higher relative area of AP1, greater relative crystallinity, and lower amylose content (Figure 1B; Table 2).

Similarly, the onset, peak, and conclusion temperatures exhibited negative correlations with amylose content [19]. This relationship arises from amylose’s interference with the organization and stabilization of granular crystals, leading to decreased resistance to gelatinization [19,29]. In addition, amylopectin molecules with relatively short branch chains may form well-aligned double helices within crystalline lamellas, requiring a higher temperature for dissociation during rice cooking [40]. In our study, gelatinization temperatures showed significant negative correlations with amylose content and significant positive correlations with most starch structural parameters, such as the relative area of AP1 and relative crystallinity (Figure 5 and Figure 6). Hence, the increased gelatinization temperatures of rice flour in three major Chinese CS_S_ under warming conditions can be attributed to the increased relative area of AP1 and relative crystallinity, along with decreased amylose content.

Protein also holds significance in determining eating quality by influencing starch gelatinization properties. Proteins serve a dual function: they compete with starch for water binding while simultaneously forming networks with amylose molecules through disulfide bonds, thereby affecting both thermal and textural characteristics [41]. For instance, Zhan et al. [42] suggested that starch granule-associated proteins, especially starch granule-associated channel proteins, may enhance the apparent crystallinity and pasting temperature while promoting the structural stability of rice starch. In our study, the protein content of head rice in three rice CS_S_ significantly increased under warming conditions and showed significant correlations with gelatinization enthalpy and temperatures (Figure 1D and Figure 5), potentially leading to elevated gelatinization enthalpy and temperatures. Therefore, the impact of protein on rice’s thermal and textural properties cannot be neglected. However, there remains limited information regarding the morphological and structural properties of protein bodies in rice kernels under warming conditions.

In summary, our multi-site field warming experiment, which simulates midcentury climate conditions, revealed significant increases in rice thermal stability and cooked rice stickiness. These changes indicate a shift in rice cooking and eating qualities across three rice cropping regions under warming conditions. However, many aspects regarding the effects of warming on rice thermal properties and cooked rice texture remain incompletely understood, especially the alterations in starch structure at multiple levels. Furthermore, the multi-level structure of rice starch is related to variety characteristics and susceptible to environmental factors [43,44]. In our study, we only conducted a preliminary exploration into the effect of warming on the crystalline structure of starch and the weight distribution of debranched starch in 2022. Therefore, future investigations necessitate multi-year experiments to isolate the impact of warming on the multi-level structure of starch and its correlation with rice quality while eliminating the influence of other factors.

## 4. Conclusions

In conclusion, our study examined the effects of warming on cooked rice textural and thermal properties in three major Chinese rice CS_S_. Overall, warming significantly elevated the stickiness of cooked rice and the gelatinization enthalpy and gelatinization temperatures of rice flour, albeit with variations observed between the two years. In addition, we observed significantly decreased amylose content, increased protein content, and changes in starch molecules and crystalline structure under warming conditions. The enhanced stickiness of cooked rice and increased rice thermal stability under warming conditions were primarily influenced by rice amylose and protein contents and starch structures. These novel insights shed light on the influence of climate warming on cooked rice texture and rice thermal properties, potentially informing strategies to enhance rice quality in the face of future climate change across three major Chinese rice CS_S_.

## Figures and Tables

**Table 1:** The *F*-values of three-way analysis of variance.

Cropping System	Source	Rice Components				Cooked Rice Texture		Thermal Properties			
		Total Starch Content (%)	Amylose Content (%)	Amylopectin Content (%)	Protein Content (%)	Hardness (g)	Stickiness (g)	Gelatinization Enthalpy (J g^−1^)	Onset Temperature (°C)	Peak Temperature (°C)	Conclusion Temperature (°C)
Single rice	Year (Y)	0.17	11.70 *	2.65	5.70	17.34 **	5.96	0.04	1.07	0.05	0.01
	Treatment (T)	0.28	13.40 *	3.40	18.36 **	3.05	8.86 *	19.75 **	22.22 **	12.49 *	9.86 *
	Y × T	0.05	3.029	0.79	0.05	0.21	0.11	0.08	3.82	1.97	0.92
Middle rice	Year (Y)	0.07	10.64 *	2.00	15.94 **	11.92 *	0.23	2.12	18.82 **	3.96	21.97 **
	Treatment (T)	0.05	6.61 *	1.25	12.23*	4.24	33.49 **	26.29 **	2.36	7.31 *	17.92 **
	Y × T	0.21	0.57	0.37	0.34	0.24	0.48	2.34	0.01	0.02	0.02
Double rice (early rice)	Year (Y)	0.40	4.88	1.34	0.07	1.06	0.59	6.62 *	0.07	4.30	3.62
	Treatment (T)	0.80	6.03 *	0.06	12.91 *	2.10	8.72 *	6.46 *	8.92 *	7.56 *	8.40 *
	Y × T	0.00	0.03	0.00	0.45	7.37 *	0.79	0.00	0.30	0.06	0.03
Double rice (late rice)	Year (Y)	0.00	0.44	0.02	0.96	6.86 *	9.13 *	9.95 *	7.75 *	1.53	7.27 *
	Treatment (T)	1.60	14.40 **	1.08	31.74 **	0.08	7.87 *	30.64 **	25.63 **	11.38 *	9.46 *
	Y × T	0.10	0.29	0.00	0.00	0.48	0.38	0.68	1.30	2.85	2.74

* and ** indicate significance at the *p* < 0.05 and *p* < 0.01 levels, respectively.

**Table 2:** Effects of experimental warming on the weight distribution parameters of debranched starch and the relative crystallinity of rice flour in three major Chinese rice cropping systems (2022).

Cropping System	Treatment	Relative Area (%)			Relative Crystallinity (%)
		AP1	AP2	AM	
Single rice	Control	59.5 b	18.3 b	20.9 a	16.8 b
	Warming	60.6 a	19.4 a	18.9 b	17.9 a
Middle rice	Control	60.7 b	19.6 a	18.4 a	16.9 a
	Warming	62.8 a	20.8 a	15.1 b	17.8 a
Double rice (early rice)	Control	63.7 a	22.5 a	12.6 a	17.1 b
	Warming	64.5 a	22.8 a	11.5 b	18.3 a
Double rice (late rice)	Control	58.5 a	21.6 b	19.9 a	16.8 b
	Warming	59.8 a	22.8 a	17.4 b	18.0 a

Control, ambient temperature treatment; Warming, whole-growth-period warming treatment. AP1, amylopectin short branch chains; AP2, amylopectin long branch chains; AM, amylose chains. Different lowercase letters indicate significant differences between treatments at the *p* < 0.05 level by Student’s *t*-test.

## Data Availability

The original contributions presented in the study are included in the article/Supplementary Material, further inquiries can be directed to the corresponding author.

## Supplementary Materials

The following supporting information can be downloaded at: https://www.mdpi.com/article/10.3390/foods13111605/s1, Figure S1: Field warming experiment sites and field actual photography in three major Chinese rice cropping systems; Figure S2. Daily mean temperature of rice canopy during rice growing season in three major Chinese rice cropping systems; Table S1: Effects of warming on rice phenology in three major Chinese rice cropping systems; Table S2. The application and management of fertilizers in three major Chinese rice cropping systems; Table S3. Daily mean temperatures during specific rice growth stages in three major Chinese rice cropping systems; Table S4. Effects of experimental warming on the total amount and components of leached materials in three major Chinese rice cropping systems (2022).
