# Supplementary material for: Experimental Warming Increased Cooked Rice Stickiness and Rice Thermal Stability in Three Major Chinese Rice Cropping Systems
Source: Foods. 2024 May 22;13(11):1605. doi: 10.3390/foods13111605 (PMC11171534; doi:10.3390/foods13111605)
Supplement: Supplementary file 1 [file foods-13-01605-s001.zip › foods-2998191-supplementary.pdf]

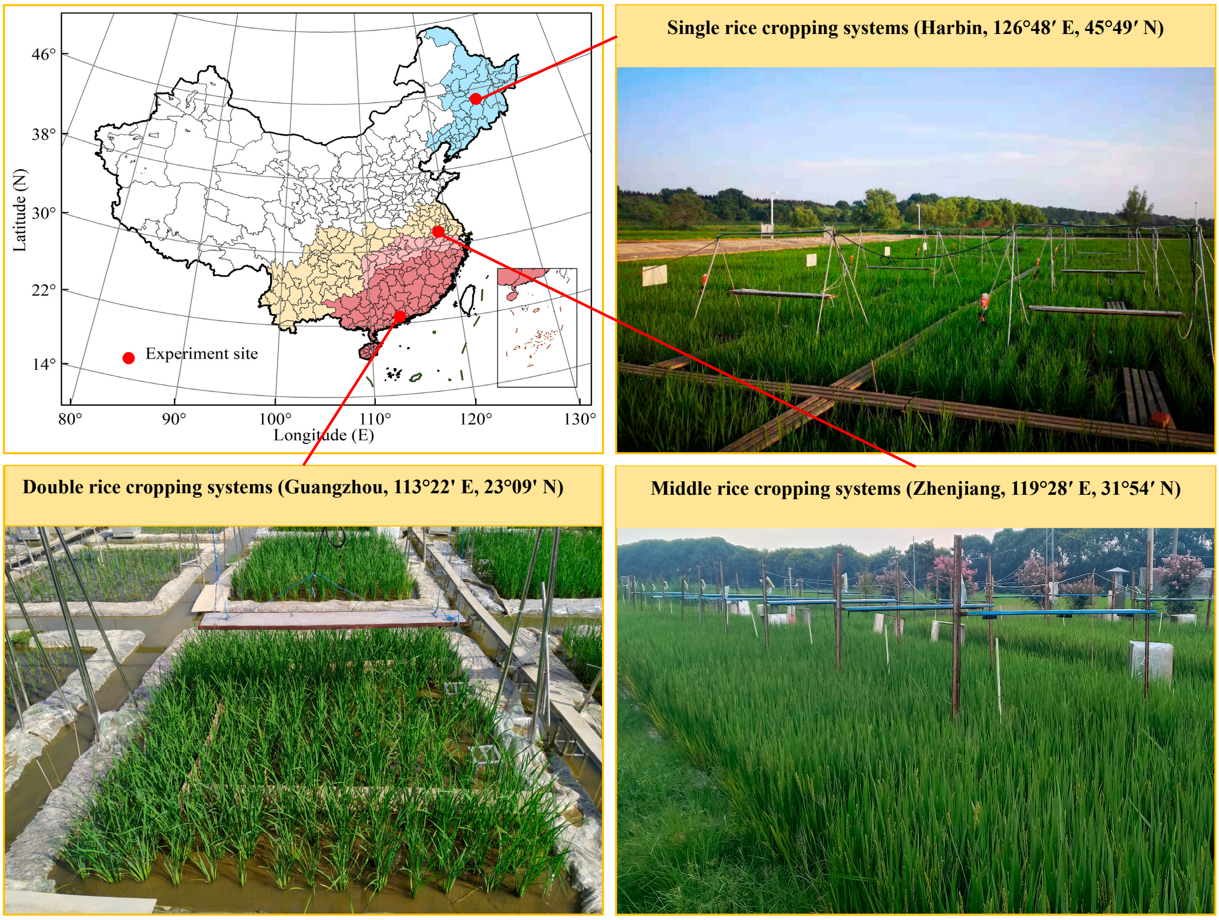

Figure S1. Field warming experiment sites and field actual photography in three major Chinese rice cropping systems. The single rice cropping systems is indicated in blue, the middle rice cropping systems is indicated in yellow, and the double rice cropping systems is indicated in red. (The light red area indicates the overlapping of middle rice cropping systems and double rice cropping systems.)

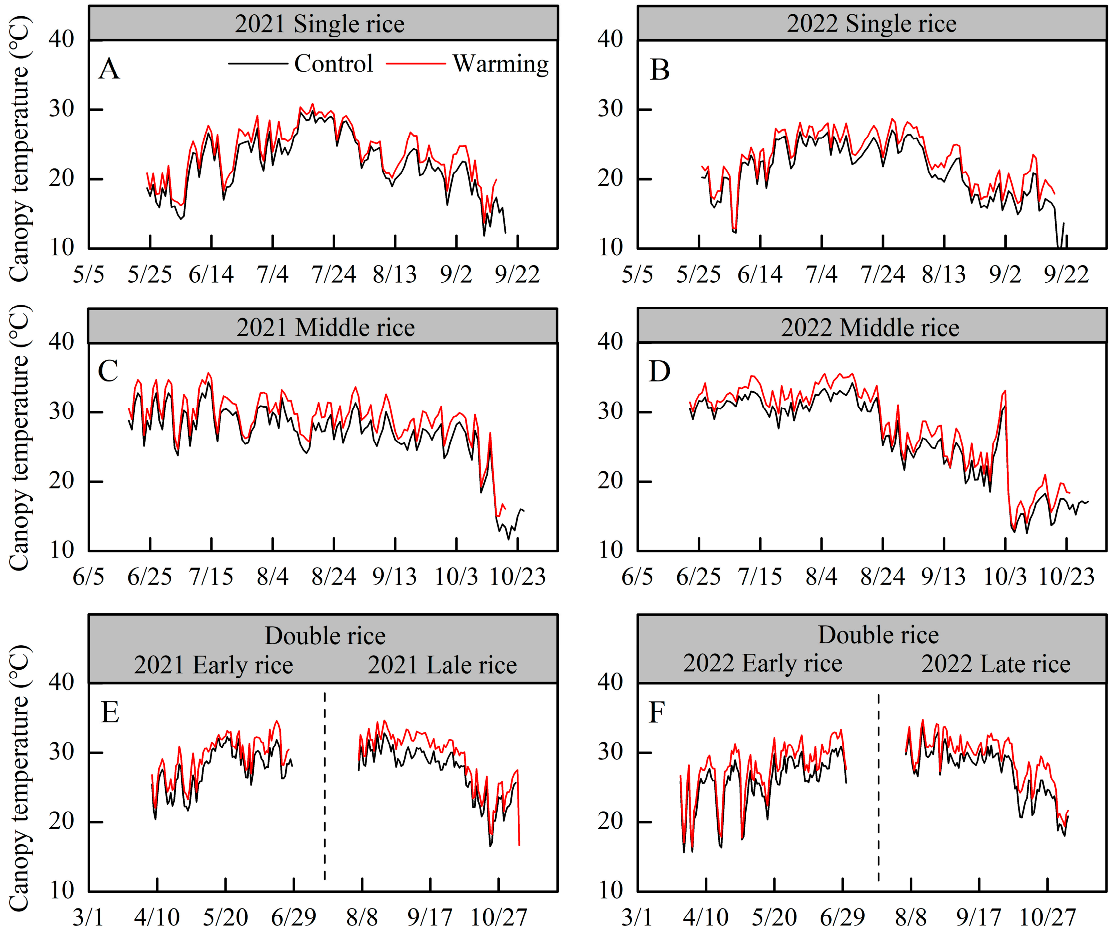

Figure S2. Daily mean temperature of rice canopy during rice growing season in three major Chinese rice cropping systems. Control, ambient temperature treatment; Warming, whole growth period warming treatment.

Table S1. Effects of warming on rice phenology in three major Chinese rice cropping systems.

Cropping system	Year	Treatment	Sowing	Transplanting	Heading	Maturity
Single rice	2021	Control	4/18	5/21	7/25	9/18
		Warming	4/18	5/21	7/20	9/15
	2022	Control	4/19	5/24	7/26	9/21
		Warming	4/19	5/24	7/22	9/18
Middle rice	2021	Control	5/28	6/17	9/8	10/26
		Warming	5/28	6/17	9/1	10/20
	2022	Control	5/31	6/21	9/10	10/30
		Warming	5/31	6/21	9/2	10/25
Double rice	2021	Control	3/16	4/6	5/29	6/29
(Early rice)		Warming	3/16	4/6	5/28	6/27
	2022	Control	2/28	3/26	5/31	7/3
		Warming	2/28	3/26	5/28	6/29
Double rice	2021	Control	7/19	8/5	9/29	11/7
(Late rice)		Warming	7/19	8/5	10/1	11/9
	2022	Control	7/19	8/3	9/27	11/8
		Warming	7/19	8/3	9/27	11/8

Control, ambient temperature treatment; Warming, whole growth period warming treatment.

Table S2. The application and management of fertilizers in three major Chinese rice cropping systems.

Fertilizer	Cropping system	Total (kg hm^{-2})	Base fertilizer (kg hm^{-2})	Tillering fertilizer (kg hm^{-2})	Panicle fertilizer (kg hm^{-2})
Nitrogen (N)	Single rice	120.0	60.0	48.0	12.0
	Middle rice	225.0	112.5	45.0	67.5
	Double rice (Early/late rice)	150.0	75.0	30.0	45.0
Phosphorus (P_2O_5)	Single rice	70.0	—	—	—
	Middle rice	180.0	—	—	—
	Double rice (Early/late rice)	48.0	—	—	—
Potassium (K_2O)	Single rice	70.0	35.0	—	35.0
	Middle rice	160.0	80.0	—	80.0
	Double rice (Early/late rice)	188.0	131.6	—	56.4

Table S3. Daily mean temperatures during specific rice growth stages in three major Chinese rice cropping systems.

Cropping system	Year	Treatment	Heading to maturity (°C)	Transplanting to maturity (°C)
Single rice	2021	Control	21.1±0.3	22.3±0.2
		Warming	23.5±0.4	24.0±0.1
	2022	Control	19.5±0.2	21.2±0.1
		Warming	22.0±0.3	23.0±0.2
Middle rice	2021	Control	23.3±0.1	26.7±0.3
		Warming	26.9±0.4	29.2±0.2
	2022	Control	19.5±0.1	25.8±0.0
		Warming	22.4±0.4	27.8±0.4
Double rice	2021	Control	28.8±0.2	27.7±0.1
(Early rice)		Warming	31.0±0.3	29.5±0.4
	2022	Control	28.2±0.2	25.7±0.2
		Warming	30.4±0.2	27.7±0.2
Double rice	2021	Control	24.0±0.3	27.2±0.1
(Late rice)		Warming	25.3±0.2	29.2±0.1
	2021	Control	24.2±0.1	27.3±0.3
		Warming	26.7±0.4	29.3±0.4

Control, ambient temperature treatment; Warming, whole growth period warming treatment.

Table S4. Effects of experimental warming on the total amount and components of leached materials in three major Chinese rice cropping systems (2022).

Cropping system	Treatment	Total leachate content (%)	Leached starch content (%)	Leached amylose content (%)	Leached amylopectin content (%)
Single rice	Control	8.69a	78.1a	17.9a	60.2a
	Warming	9.36a	77.9a	18.5a	59.4a
Middle rice	Control	12.17a	79.1a	17.8a	61.3a
	Warming	12.14a	80.6a	18.9a	61.7a
Double rice (Early rice)	Control	13.78a	77.9a	16.6a	61.3a
	Warming	14.01a	75.7a	15.6a	60.2a
Double rice (Late rice)	Control	12.48a	80.7a	19.6a	61.1a
	Warming	12.74a	79.2a	19.0a	60.2a

Control, ambient temperature treatment; Warming, whole growth period warming treatment.
